# A characteristic mode analysis-based isolation enhancement for a THz MIMO antenna

**DOI:** 10.1038/s41598-026-48109-x

**Published:** 2026-04-11

**Authors:** Praveen Kumar

**Affiliations:** Manipal Institute of Technology, Manipal Academy of Higher Education, Manipal, India

**Keywords:** THz antenna, Hybrid decoupling structure, MIMO, Neutralization line, Wideband, SDG9: Industry, Innovation and infrastructure, Engineering, Physics

## Abstract

The terahertz (THz) frequency range has attracted research interest due to its wide available spectrum and short wavelengths. This work provides a comprehensive analysis of an wideband THz multiple input and multiple output (MIMO) antenna that uses a neutralization line (NL) to reduce inter-element interference. A standard circular patch antenna is modified by reducing the ground plane to create a wideband THz antenna. Furthermore, a four-port THz MIMO antenna is produced by horizontally and vertically replicating the designed THz antenna with a size of 46 × 46 × 2 µm^3^. A hybrid decoupling structure consisting of a transformed ground plane and a rectangular-shaped neutralization line reduces the inevitable mutual coupling between the MIMO elements. The proposed THz MIMO antenna exhibits isolation greater than 22 dB over the operational frequency range of 3.3–11.1 THz. The proposed THz MIMO antenna is analyzed for radiation properties and diversity features, such as ECC < 0.1, DG > 9.5 dB, MEG≤-6 dB, TARC < -10 dB, CCL < 0.2 bps/Hz, and ME < 0 dB. The results validate the effectiveness of the CMA-based isolation enhancement framework within the investigated THz frequency range under full-wave electromagnetic simulation.

## Introduction

Wireless data transmission has increased over the past two decades due to the advancement in electronic devices and technology. Electronic device applications such as online gaming, high-definition video, voice, and data transfer in 5G demand high channel capacity as they involve massive uploading and downloading of data. The high data requirement for current and future technologies is fulfilled by the terahertz (THz) bands. THz bands range in frequency from 0.1 THz to 30 THz, with a bandwidth of hundreds of GHz. The wide available spectrum in the THz regime theoretically enables extremely high data rate potential^1–5^. Reduced wavelength results in physically smaller antenna dimensions; however, electrical compactness remains frequency-dependent. Having several benefits, fading as a result of signal dispersion and reflections remains a significant issue^6–8^. Often, fading affects the channel, which makes THz devices function worse. More radiating components can be employed in order to address the fading problem and enhance the performance of the THz communication system. A compact multiple input and multiple output (MIMO) antenna may be constructed due to the ability of these compact antennas to produce many more numbers in a given physical space. Therefore, in a multipath-rich environment, faster data rates, enhanced channel capacity, and reliable communication may be attained^9,10^.

A variety of design approaches for THz single, array configurations, and MIMO patch antenna structures have been incrementally established by researchers. Using the conventional rectangular patch and metamaterial (MTM) loading, the THz antenna is constructed and extensively studied. The authors showed that impedance bandwidth is enhanced by MTM loading on the radiator. Conversely, the radiation characteristics are enhanced when the MTM surface is loaded onto the radiator^11^. A leaf-shaped radiator and curved reduced ground plane monopole antenna operating in the wideband frequency of 130% impedance bandwidth are presented in^12^. Further, the THz antenna is configured orthogonally to form a MIMO antenna with a common ground plane with isolation better than 18 dB. As seen in^13^, the rectangular patch antenna radiator is defective due to the use of parasitic components with a smaller ground plane to realize the wider impedance bandwidth THz MIMO antenna. Using a defective ground plane (DGP), the inter-element isolation is achieved better than 20dB over the operational frequency. A comprehensive analysis of the graphene-based microstrip line-driven slot ring THz MIMO antenna operating in 5.68–6.51 THz is given in^14^. To increase the isolation in the operating frequency of the antenna, the presented THz MIMO antenna employs orthogonal orientation, DGP, and a rectangular stub between the elements onto the radiator. In order to develop a THz MIMO antenna, a rectangular split ring resonator MTM unit is etched on the rectangular patch with a decreased ground plane shown in^15^. Although the MTM unit etching offers a greater impedance bandwidth, the antenna that is being shown has inadequate isolation at the center frequency. The work presented in^16^ is a unique, compact, scalable multi-port antenna that can function in several bands at THz frequencies. The arrangement of the two-port antenna construction provides excellent isolation between the ports without the need for a decoupling device. Additionally, this study has investigated the scalability of the number of elements in the MIMO system while maintaining the antenna’s multi-band feature and diversity performance. The filter on a silicon substrate antenna of the dielectric resonator category is used to create a THz MIMO antenna and confines its isolation among the ports. The antenna’s ground plane is modified by introducing the parasitic element, resulting in improved isolation of the THz MIMO antenna^17^. A two-port shared slotted radiator structure working in the dual-band is proposed in^18^. The isolation among the ports is improved by inserting the elliptical and circular slots into the shared radiator. In^19^, an octagon-shaped parasitic element loaded with the decreased ground plane is shown to function at sub-THz frequencies. Further antennas are arranged orthogonally to produce the MIMO antenna, which has an isolation of greater than 18 dB. A quad-band THz MIMO using a modified rectangle as an S-shape radiator with a reduced ground plane is presented in^20^. A T-shape rectangular slotted radiator is orthogonally configured to create a four-port MIMO antenna operating in a sub-THz frequency range. Because of the orthogonal orientation, the elements are more isolated from one another^21^. A spiral-shaped antenna is replicated vertically to form a MIMO antenna operating above 10 THz frequency. The ground plane of the THz MIMO antenna is modified by introducing the parasitic elements. The DGP of the antenna distracts the uniform current flow, creates local current channels, and helps in realizing the required mutual coupling^22^. A metamaterial-based fractal two-port THz MIMO antenna with a decreased ground plane is presented in^23^. The reported antenna here demonstrates larger dimensions and narrow operating bandwidth, and in MIMO, some of the antenna designs shortfall the shared ground plane. The inter elements are decoupled with each other using orthogonal configuration and DGP.

This work presents a modified circular patch wideband THz MIMO antenna with total dimensions of 46 × 46 × 2 µm^3^. The suggested antenna provides isolation greater than 22 dB across an operational frequency range of 3.3–11.1 THz. A rectangle-shaped neutralization line (NL) dissociates the impact of inter-element interaction. The suggested antenna is evaluated for scattering parameters by comparing results to the antenna’s corresponding equivalent circuit, radiation properties, and diversity features. The primary findings of this study include the development of a CMA-based THz MIMO antenna design approach, operation over 3.3–11.1 THz, isolation exceeding 22 dB across the impedance bandwidth, and stable multi-port diversity characteristics under full-wave electromagnetic simulation. The present study focuses on electromagnetic and modal analysis of the antenna structure; system-level propagation and communication modeling are beyond its scope. The remainder of the article is as follows: Sect.  Design methodology of wideband THZ MIMO antenna depicts the comprehensive design strategy for the THz MIMO antenna and explains how the decoupling structure evolved. Section  Results and discussion presents the results of the proposed antenna. Section  Conclusion has concluding observations.

## Design methodology of wideband THZ MIMO antenna

**Fig. 1:**
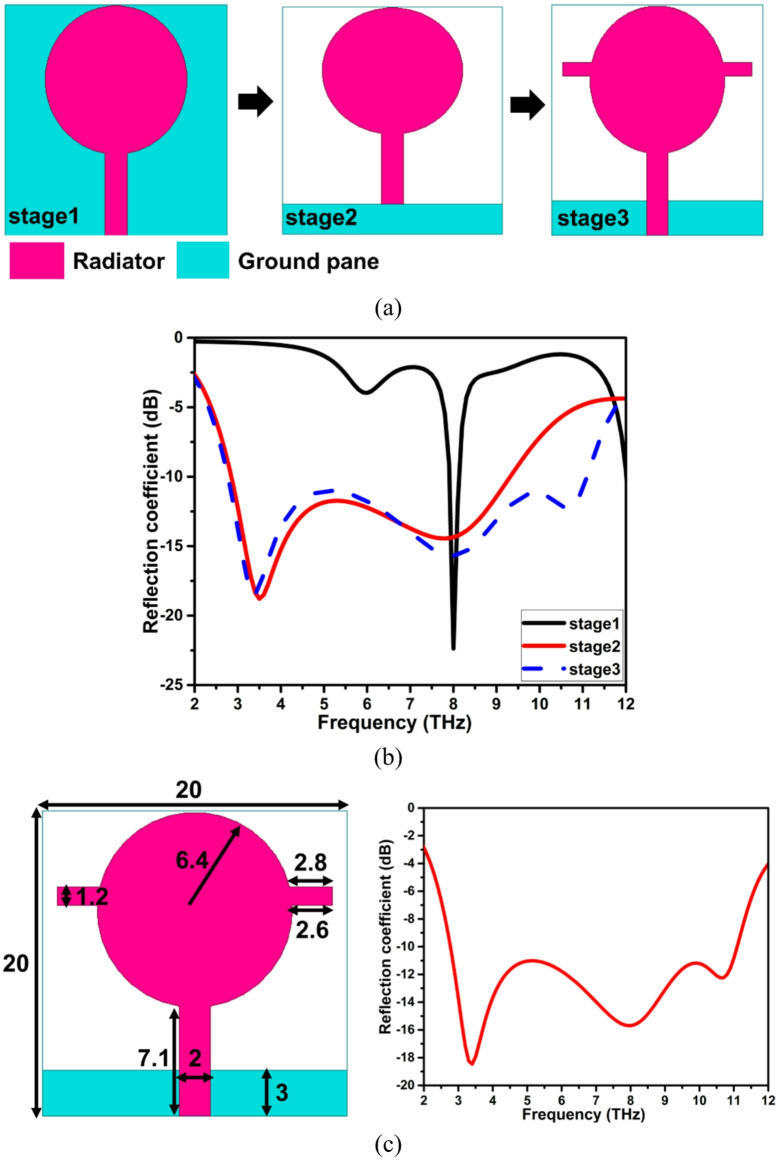
Wideband THz antenna (**a**) antenna evolution, (**b**) reflection coefficient of the antenna evolution, (**c**) geometry and reflection coefficient of the proposed antenna (dimensions are in µm).

The THz antenna is realized by sequentially modifying the conventional circular patch, as depicted in Fig. 1a. The ground plane of the circular patch is lowered, causing the lumped parameters to change, resulting in a lower quality factor and a higher bandwidth. Further, to improve the impedance bandwidth of the antenna, particularly towards the lower side, additional conducting material is incorporated into the radiator. The increased dimension of a patch is inversely proportional to the bandwidth, which helps in achieving a wider impedance bandwidth, as depicted in Fig. 1b. The designed wideband THz antenna functions from 2.8 to 11.1 THz, having a polyamide substrate (dielectric constant of 4.3 and loss tangent of 0.0027 ) of dimension 20 × 20 × 2 µm^3^. The polyimide substrate was chosen because it offers unique properties such as a low dielectric constant, low loss tangent, high thermal stability, and mechanical flexibility, making it perfect for THz frequencies, particularly in wearable, flexible, or conformal antennas and sensors. Substrates such as silicon and GaAs are commonly used for high-precision or on-chip applications, but they lack the versatility required for flexible designs. Teflon and quartz have low losses but lack flexibility and ease of integration. The geometrical parameter values and the reflection coefficient curve of the THz antenna are represented in Fig. 1c.

### Parametric analysis

**Fig. 2:**
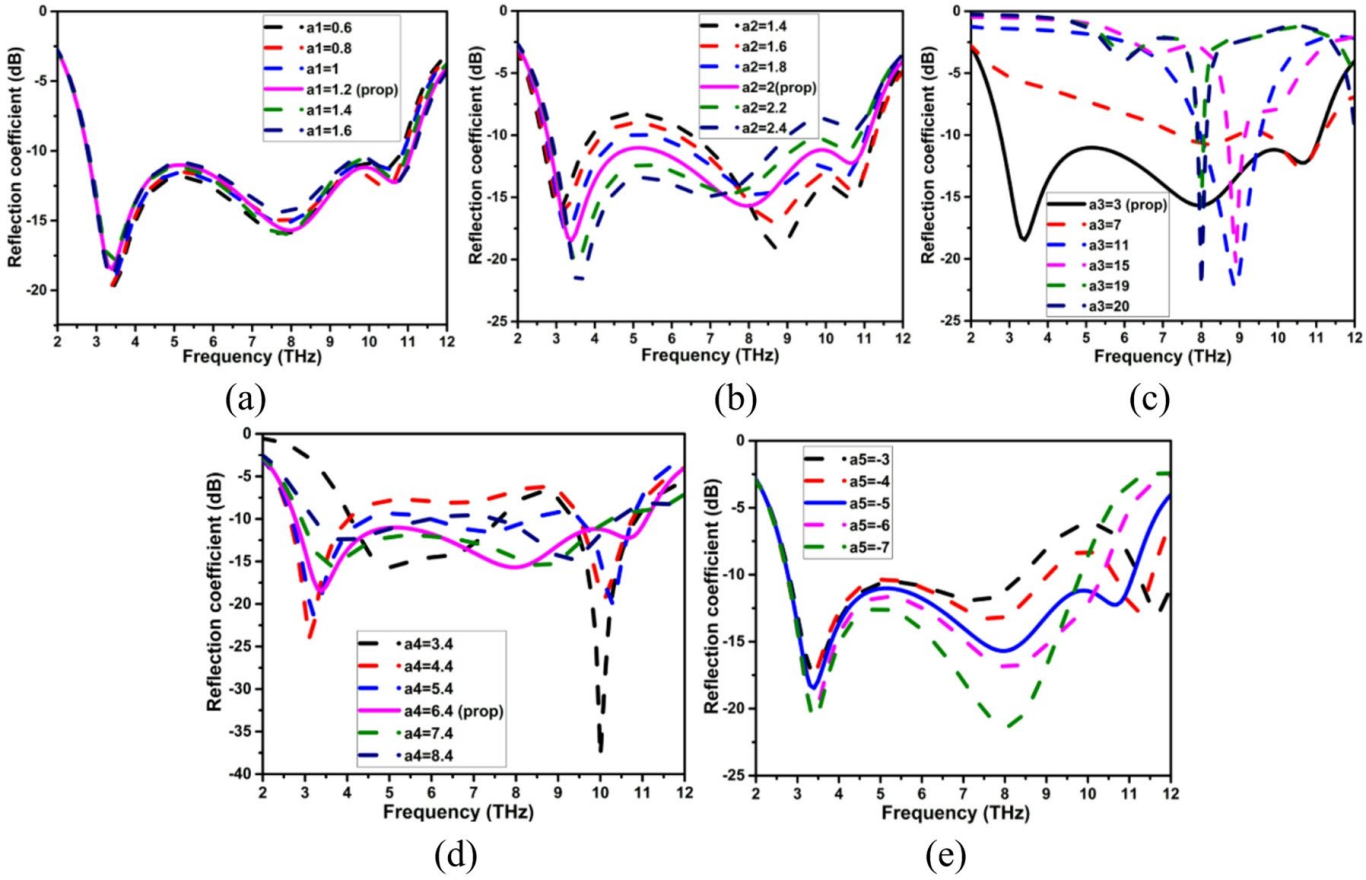
Parametric analysis of the proposed THz antenna (**a**) arm width (a1), (**b**) feed width (a2), (**c**) ground plane (a3), (**d**) radius of a patch (a4), and (**e**) position of the arm (a5) (dimensions are in µm).

An antenna’s performance is examined by changing various physical parameters and observing how those changes impact the antenna’s properties, such as impedance matching, bandwidth, resonant frequency, and radiation properties. This process is known as a parametric analysis of the antenna. Antennas are essential parts of wireless communication systems, and understanding how they behave under various circumstances aids in optimizing the design of the antenna for specific uses. Wireless communication systems require antennas, and knowing how they function in different situations helps designers make the best possible antenna design. In this case, antenna geometry and design parameters are varied in a sequential step size to realize the wider impedance bandwidth through impedance matching, as depicted in Fig. 2. As shown in Fig. 2a, the arm width (a1) is progressively changed from 0.6 to 1.6 μm with 0.2 μm variation. The modification of a1 has only a little effect on the impedance bandwidth. Similarly, the geometrical parameters feed width (a2), ground plane (a3), radius of the patch (a4), and position of the arm (a5) are adjusted, and the resulting reaction is shown in Fig. 2(b–e). The incremental modification of a2 pushes the lower resonance frequency slightly upward. The lowered ground plane (a3) disrupts the uniform current distribution and contributes to the realization of a larger impedance bandwidth. Variations in parameters a4 and a5 considerably impact the impedance bandwidth. The ideal values of these parameters are chosen as a1 = 1.2 μm, a2 = 2 μm, a3 = 3 μm, a4 = 6.4 μm, and a5 = -5 μm, respectively.

### Two port wideband THz antenna

**Fig. 3:**
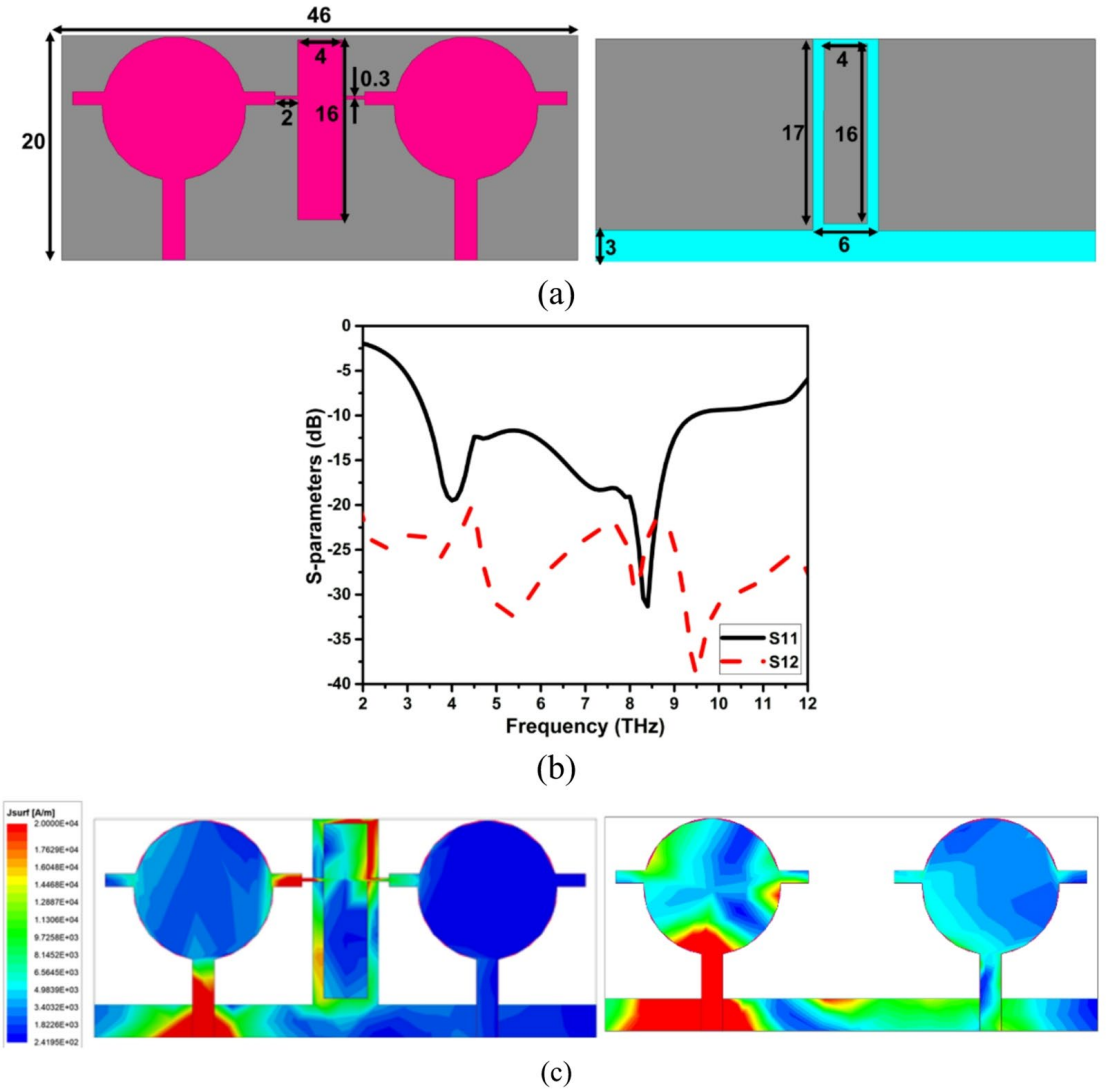
The proposed THz MIMO antenna (**a**) Geometrical details, (**b**) S-parameters. (**c**) Surface current distribution with and without decoupling structure.

By reproducing horizontally with an edge to edge separation of 8 μm, the designed wideband THz antenna is changed into two port MIMO. The presented antenna has a substrate dimension of 20 × 46 × 2 µm^3^, as represented in Fig. 3a. The inevitable mutual coupling between the interelement is reduced by introducing a hybrid decoupling structure comprised of NL and DGP. A rectangular parasitic element is connected to the edges of the two antenna elements that form NL and help in reducing the mutual coupling. Further isolation is enhanced by combining the DGP to NL, a hybrid decoupling structure that provides isolation of better than 23 dB across the impedance bandwidth of 3.4-11THz, as depicted in Fig. 3b. The NL produces the field precisely out of phase with the same magnitude as that of the excited antenna element. Therefore, coupling among the elements cancels and aids in decreased mutual coupling. Figure 3c demonstrates the effectiveness of the decoupling structure compared to the structure without decoupling for the isolation improvement of the proposed THz MIMO antenna design.

### Decoupling structure evolution

**Fig. 4:**
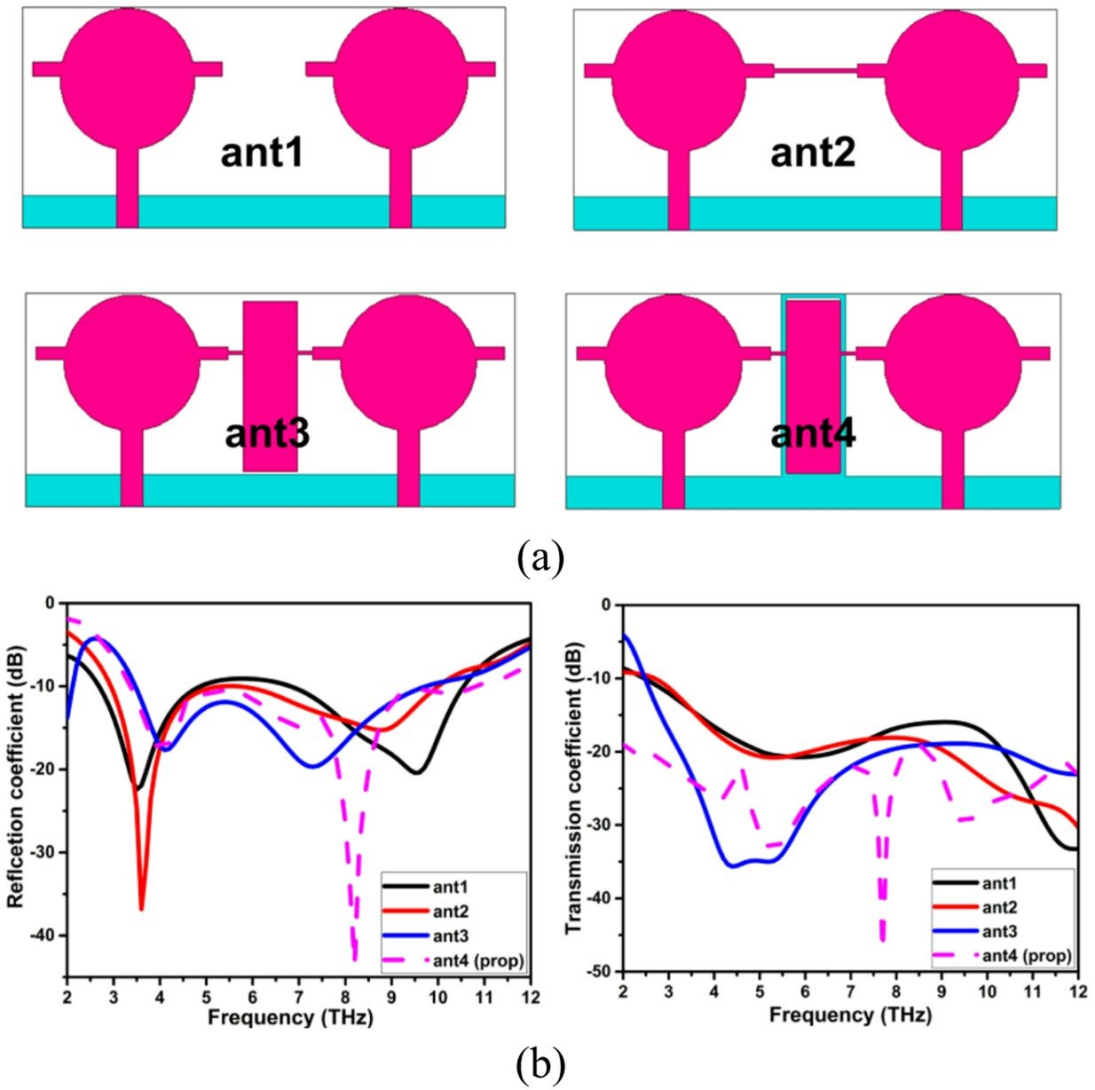
Decoupling structure evolution of THz MIMO antenna (**a**) Evolution, (**b**) S-parameters of the evolution.

The development of the two-port THz antenna’s decoupling structure and its S-parameters are depicted in Fig. 4. Figure 4(a, b) illustrates how the ant1 design offers less isolation towards the lower frequency. In order to create a hybrid decoupling structure with isolation greater than 22 dB over the impedance spectrum, NL in the shape of a rectangle and DGP are joined. The characteristic mode analysis is used to study the evolution of the decoupling structure in detail.

**Fig. 5:**
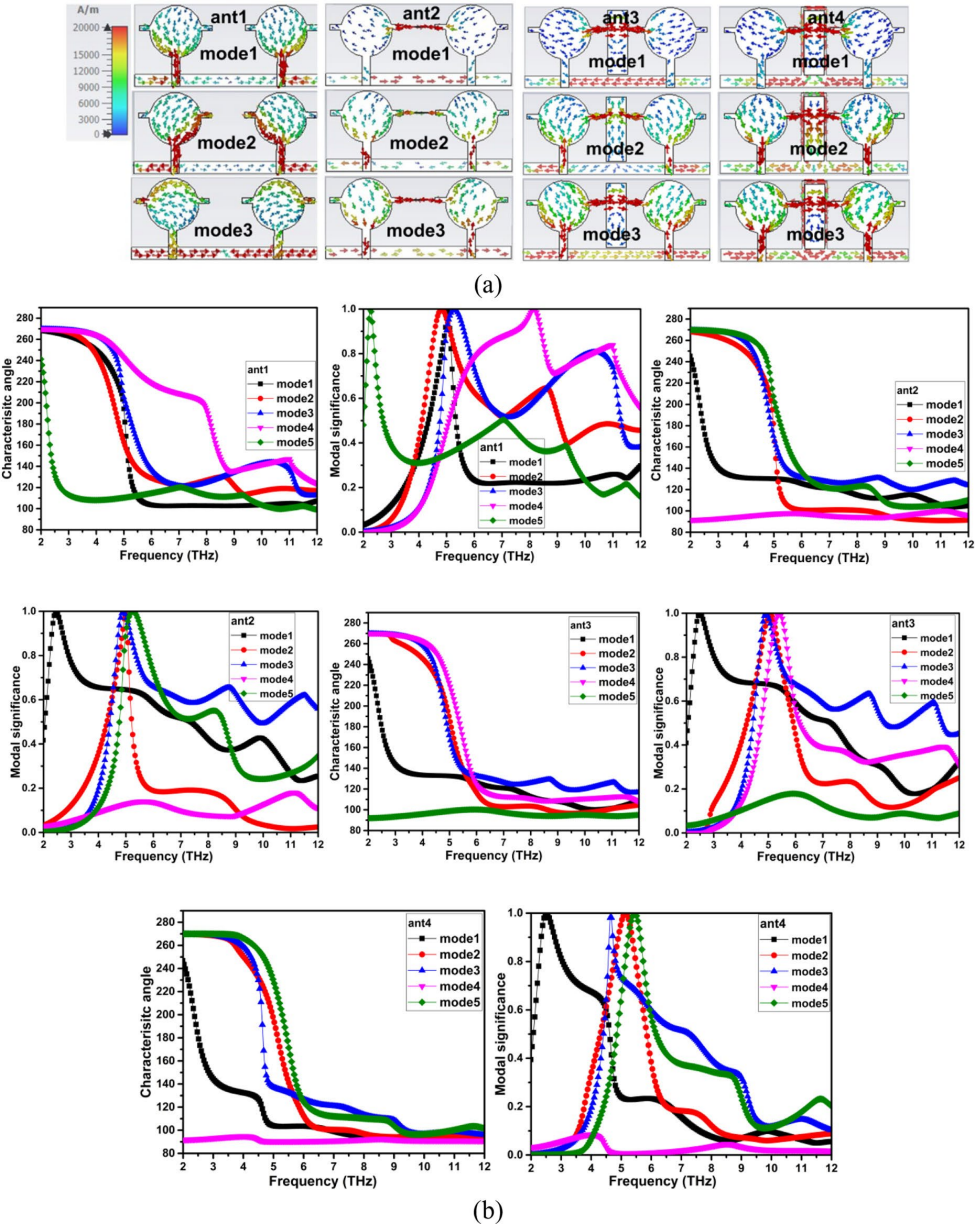
Character mode analysis of the two port THz MIMO antenna (**a**) current distribution plot at different modes of the decoupling structure evolution, (**b**) characteristic angle and modal significance of the decoupling structure evolution.

The ideal placement of the parasitic elements on the radiator and ground plane is ascertained by analysing the surface current plots of the different modes that are simulated using the Eigen solver. An eigen solver for different modes is used to simulate the different evolutionary stages, such as ant1, ant2, ant3, and ant4, as seen in Fig. 5. For every mode in every evolutionary step of the antenna, plots of the surface vector current distribution are displayed. To apply the NL and DGS, the radiator section with the highest current concentration is selected. As can be seen in ant2 of Fig. 5a, a horizontal stub is inserted between the radiators as a result of the ant1 in Mode 2 having the highest current concentration on the radiator arm. Similarly, a hybrid decoupling framework is created by combining NL and DGS. In sync with the excited element, the NL produces an out-of-phase field, and the DGS adjusts the uniform current distribution and serves as a band stop filter. These characteristics help to provide better separation between the working frequencies of the antenna. The characteristic angle (CA) and modal significance (MS) of the two-port THz MIMO antenna, which offers a characteristic mode-based explanation of the electromagnetic behaviour of this antenna, are shown in Fig. 5b. The CA curves indicate that the most important characteristic modes overlap or are near 180 degrees in the desired operating band of THz, which is characteristic of modal resonance and efficient operation according to characteristic mode theory. Conversely, the non-dominant modes are at characteristic angles well out of 180 degrees, which validates that they are not excited strongly; hence, they do not play a significant role in radiation or coupling. The next behaviour is supported by the MS response, which shows that the dominant modes have MS values close to 1 over the resonating frequency, indicating good modal excitation and power radiation efficiency. The frequencies of higher-order modes exhibit significantly lower MS values, which represents their inhibited involvement depending on the antenna structure and decoupling structure. Together, the two modes, matching the CA minima with MS maxima, demonstrate controlled modal excitation and stable wideband operation across operating frequencies.

### Four port wideband THz antenna

**Fig. 6:**
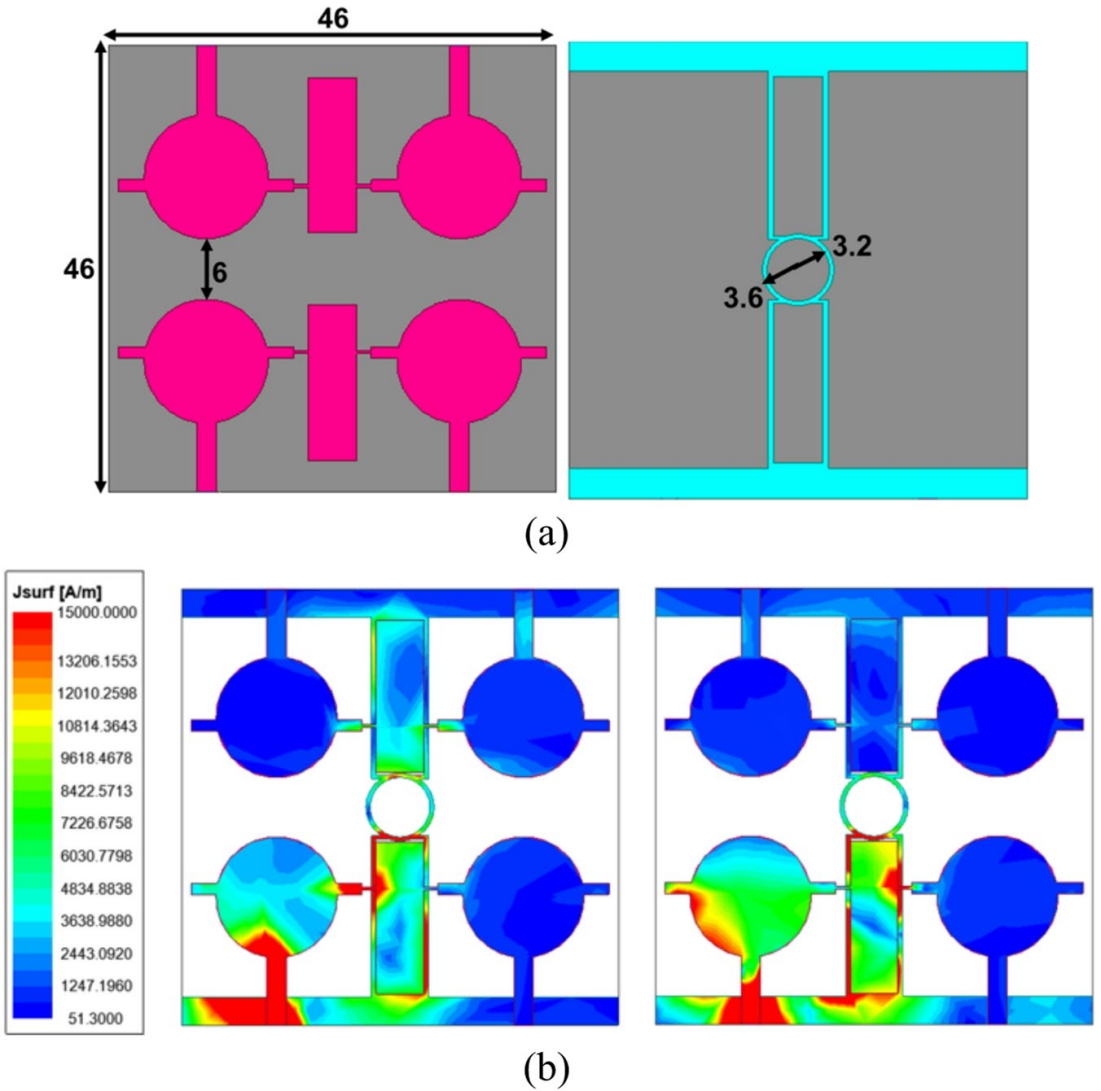
The wideband THz MIMO antenna (**a**) geometry information, (**b**) current distribution plot at 4 THz and 7.7 THz.

The proposed four port THz MIMO antenna is designed by vertically replicating the two port THz antenna with a separation of 6 μm, as represented in Fig. 6a. The substrate dimension of the proposed antenna is 46 × 46 × 2 µm^3^, operating in the frequency range of 3.3–11.1 THz with the resonance frequency of 4 THz and 7.7 THz. The hybrid decoupling structure provides an isolation of better than 23 dB across the impedance bandwidth. The same can be visualized by the current distribution plots at 4 THz and 7.7 THz, as depicted in Fig. 6b. Port 1 is excited, and other ports are terminated to study the field influence of the excited element on the neighbour one. From Fig. 6b, it can be observed that the highest current is concentrated across the centre of the patch, feedline, and onto the hybrid decoupling structure.

## Results and discussion

The behaviour of the proposed wide bandwidth THz MIMO antenna is comprehensively analyzed by characterizing its scattering parameters, equivalent circuit, radiation pattern, and gain. The corresponding RLC circuit of the proposed antenna is designed in order to verify the simulated S-parameters of the antenna.

### S-parameters

**Fig. 7:**
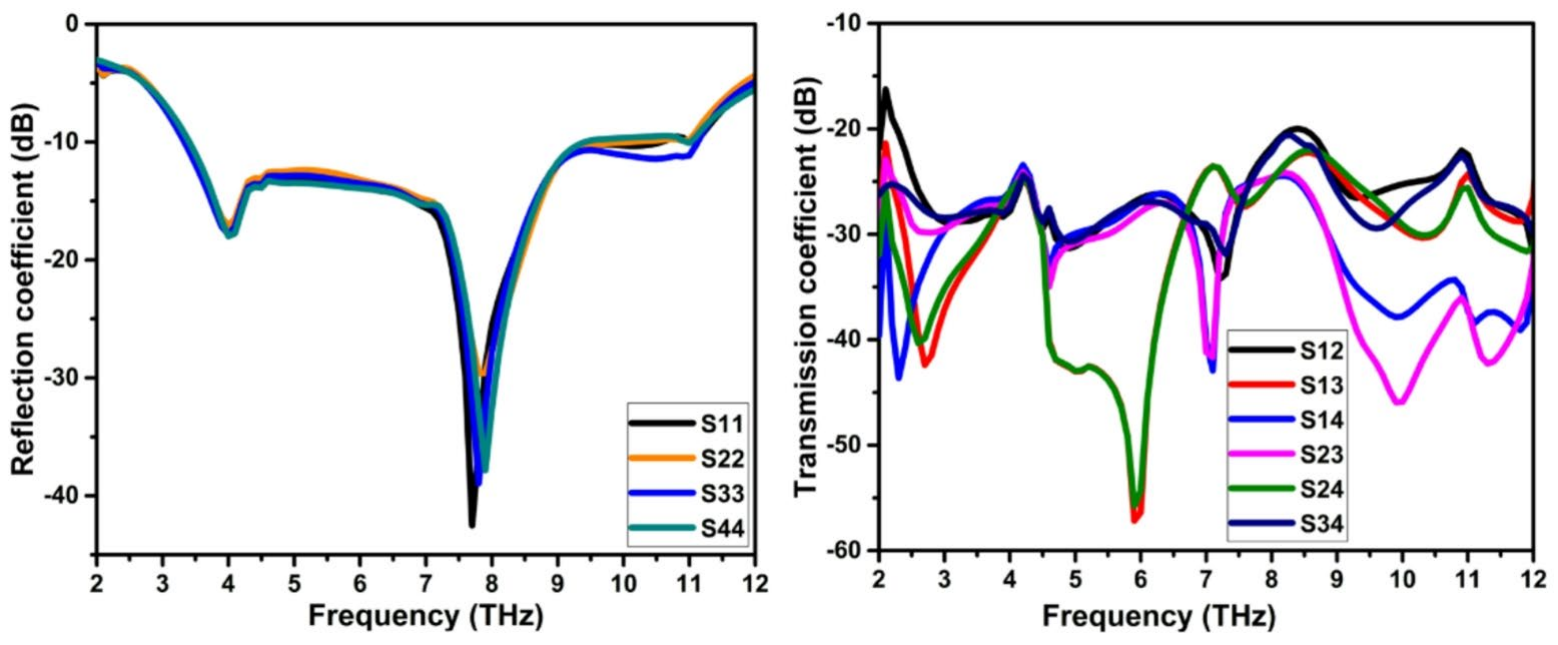
S-parameters of the wide bandwidth THz MIMO antenna (**a**) reflection coefficient, (**b**) transmission coefficient.

The simulated reflection coefficient curve of the suggested THz MIMO antenna is represented in Fig. 7a. The antenna has an impedance bandwidth of 3.3–11.1 THz. The field interaction between the elements due to the proximity is decoupled using the NL and DGP, as the decoupling structure provides isolation better than 22 dB, as depicted in Fig. 7b.

**Fig. 8:**
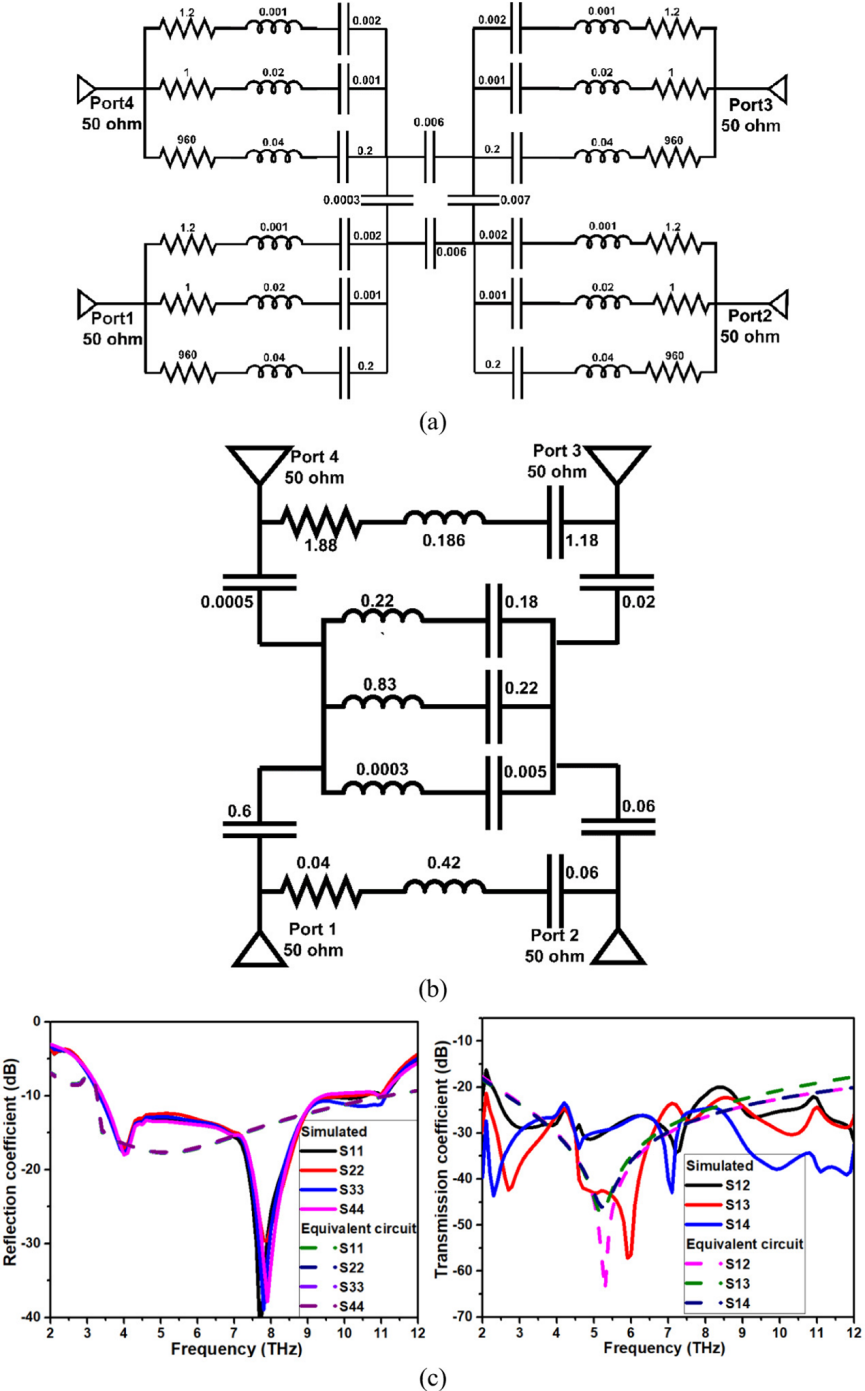
Analogous RLC circuit of proposed wide bandwidth THz MIMO antenna (**a**) radiator (**b**) ground plane, and (**c**) S-parameters.

An equivalent circuit of the proposed THz MIMO antenna is constructed using the lumped parameters resistors, inductors, and capacitors, as shown in Fig. 8. Circuit theory analysis and the impedance approach are used in the circuit’s design. The method described in^24^ is first used to calculate the values of the lumped elements. The optimal values of the components are chosen by performing the tuning and optimization technique in AWR software. The series RLC are linked in parallel to achieve the same reflection coefficient as the antenna element based on the resonance of the single element antenna. In order to synchronize the resonances in parallel configuration, the antenna resonances were collectively modeled using a series of RLC parallel-fed components. This was done since the antenna impedance represents real and imaginary components with parallel resonances that are constant within the wide bandwidth frequency of operation. Due to the symmetry of the antennas, the same circuit is replicated to form the analogous circuit of the THz MIMO antenna by exciting the circuit with the 50-ohm ports and interlinking with the capacitor, as presented in Fig. 8a. The decoupling structure’s NL is represented as series RLC, and DGP is represented as a combination of inductor and capacitor, as represented in Fig. 8b, and associated S-parameters are represented in Fig. 8c. To validate the proposed equivalent circuit model, quantitative error analysis was performed by digitizing the simulated and circuit-based S-parameter responses. The reflection coefficients exhibit excellent agreement with an average RMSE below 1.3 dB and a maximum deviation less than 3.2 dB over the entire 2–12 THz band. The transmission coefficients show slightly higher deviation near deep isolation nulls, with an average RMSE of 1.9 dB, which is attributed to the high-Q resonant nature of the decoupling structure. Overall, the strong correlation (R² > 0.97) confirms the validity of the proposed equivalent circuit model.

### Radiation properties

**Fig. 9:**
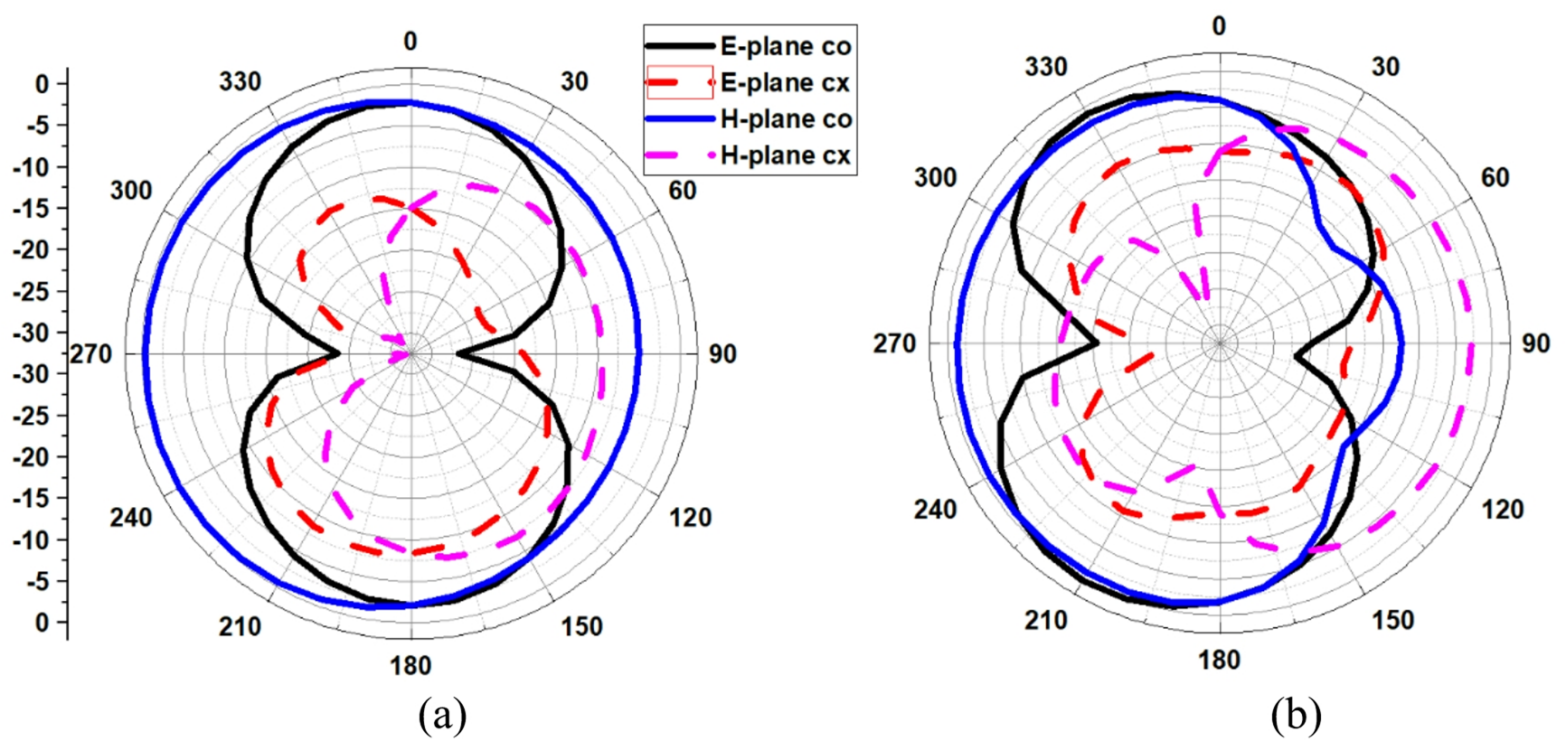
Radiation plot at (**a**) 4 THz and (**b**) 7.7 THz.

The energy emitted into space is represented by the antenna’s radiation pattern. Plotting of the co and cross-radiation field patterns in relation to the E- and H-fields was done in Fig. 9. The radiation graphs show the bidirectional and omnidirectional radiation patterns in the two major planes, E and H, at the resonant frequencies of 4 THz and 7.7 THz, respectively as depicted in Fig. 9(a, b). The bidirectional radiation behavior indicates symmetric coverage along opposite directions within the investigated THz band under full-wave simulation.

### MIMO diversity characteristics

The MIMO antenna system’s performance is determined by its diversity properties. The envelope correlation coefficient (ECC) refers to the relationship between two signal envelopes. The envelope of a signal is the amplitude of an analytical signal obtained using envelope detection. Antennas in MIMO systems are placed apart to take advantage of spatial diversity, which helps battle fading and improves system stability. The ECC determines how effectively signals received by different antennas retain their diversity properties. A low ECC is a good thing for spatial diversity since it means that the amplitudes of the signals that the antennas receive are uncorrelated. The ECC is typically computed using the 3D radiation pattern as represented in Eq. 1.

1 $$\:ECC\:\:=\frac{{\left|{\int\:}_{0}^{2\pi\:}{\int\:}_{0}^{\pi\:}\left[XPR\cdot\:{E}_{\theta\:i}{E}_{\theta\:j}^{\mathrm{*}}{P}_{\theta\:}+{E}_{\phi\:i}{E}_{\phi\:j}^{\mathrm{*}}{P}_{\phi\:}\right]\right|}^{2}}{{\int\:}_{0}^{2\pi\:}{\int\:}_{0}^{\pi\:}\left[XPR\cdot\:{E}_{\theta\:i}{E}_{\theta\:i}^{\mathrm{*}}{P}_{\theta\:}+{E}_{\phi\:i}{E}_{\phi\:i}^{\mathrm{*}}{P}_{\phi\:}\right]d\varOmega\:\:\times\:{\int\:}_{0}^{2\pi\:}{\int\:}_{0}^{\pi\:}\left[XPR\cdot\:{E}_{\theta\:j}{E}_{\theta\:j}^{\mathrm{*}}{P}_{\theta\:}+{E}_{\phi\:j}{E}_{\phi\:j}^{\mathrm{*}}{P}_{\phi\:}\right]d\varOmega\:\:}$$

where cross polarization represents XPR, E is electric field in θ and Φ.

**Fig. 10:**
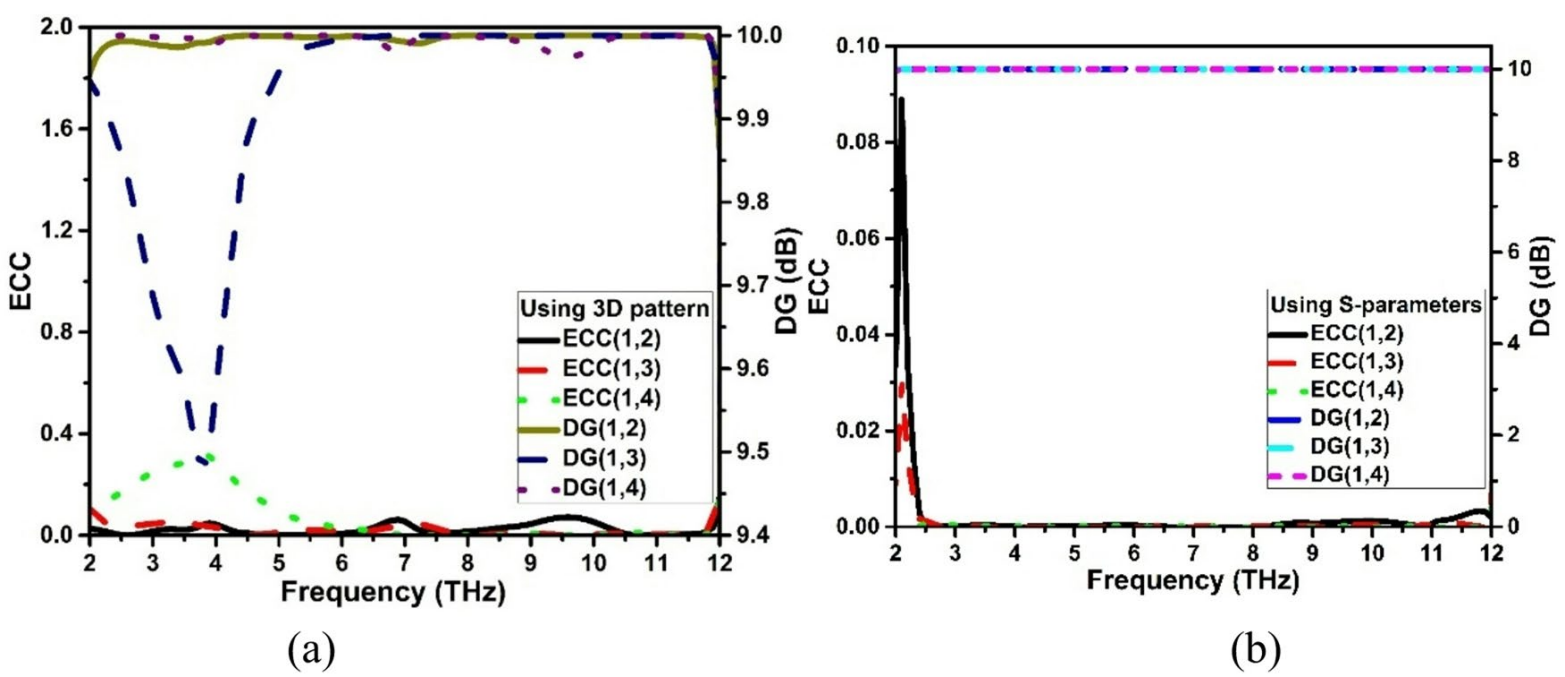
Diversity features: ECC and DG (**a**) 3D pattern (**b**) S-parameters.

In a MIMO system, better diversity gain (DG) is proportional to a low ECC. The inference is that distinct antennas receive signals that fade independently, improving the system’s resistance to fading-related signal loss. The DG is computed using Eq. 2. The ECC and DG of the suggested antenna are displayed using S-parameters and a 3D radiation pattern in Fig. 10. The computation of ECC and DG using 3 D radiation pattern is shown in Fig. 10a and using S-parameters shown in Fig. 10b.

2 $$\:DG=10\sqrt{1-ECC}$$

The mean power of an antenna received in relation to the mean power received by an isotropic antenna is measured by the mean effective gain (MEG). Ideally, the MEG should be less than or equal to -6 dB for four port antenna configuration, and the MEG ratio should be close to 0. MEG describe the overall performance of a MIMO system in terms of transmit power efficiency and is computed using Eq. (3).

3 $$\:{MEG}_{i}=0.5\:{\eta\:}_{i,rad}\:=\:0.5\left(1-\sum\:_{j=1}^{P}\left|{S}_{ij}\right|\right)\:\:\:\:\:\:\:\:$$

**Fig. 11:**
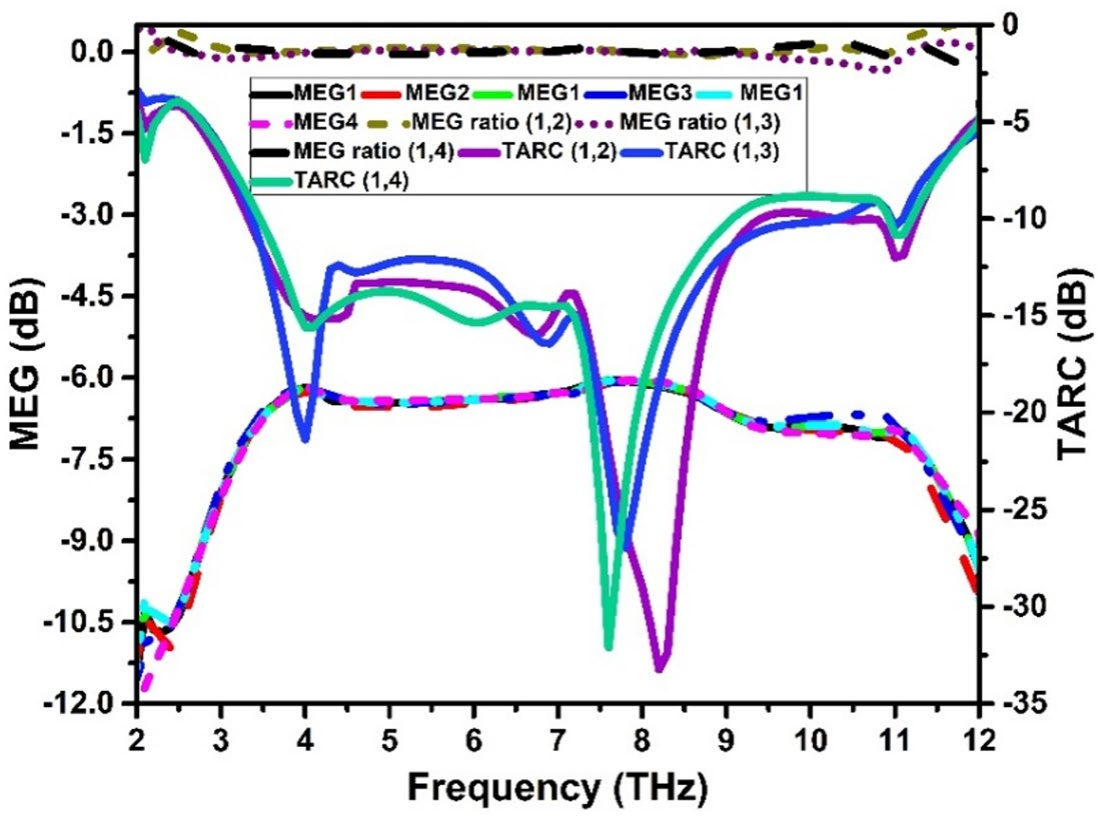
Diversity features: MEG and TARC.

When multiple antennas are utilized in transceivers, an antenna design’s effectiveness in terms of radiating power is evaluated by the total active reflection coefficient (TARC), which should be less than − 10 dB. The MIMO antenna system’s impedance matching is computed using TARC. It determines the quantity of power that the antennas reflect back to the transmitter. The TARC for a MIMO system may be represented mathematically as in Eq. (4). The MEG and TARC of the suggested antenna are displayed in Fig. 11.

4 $$\:TARC=\:\:\:\frac{\:\sqrt{\sum\:_{j=1}^{4}{\left|{S}_{j1}+\sum\:_{m=2}^{4}{S}_{jm}{e}^{j{\theta\:}_{m-1}}\right|}^{2}}}{\sqrt{4}}$$

**Fig. 12:**
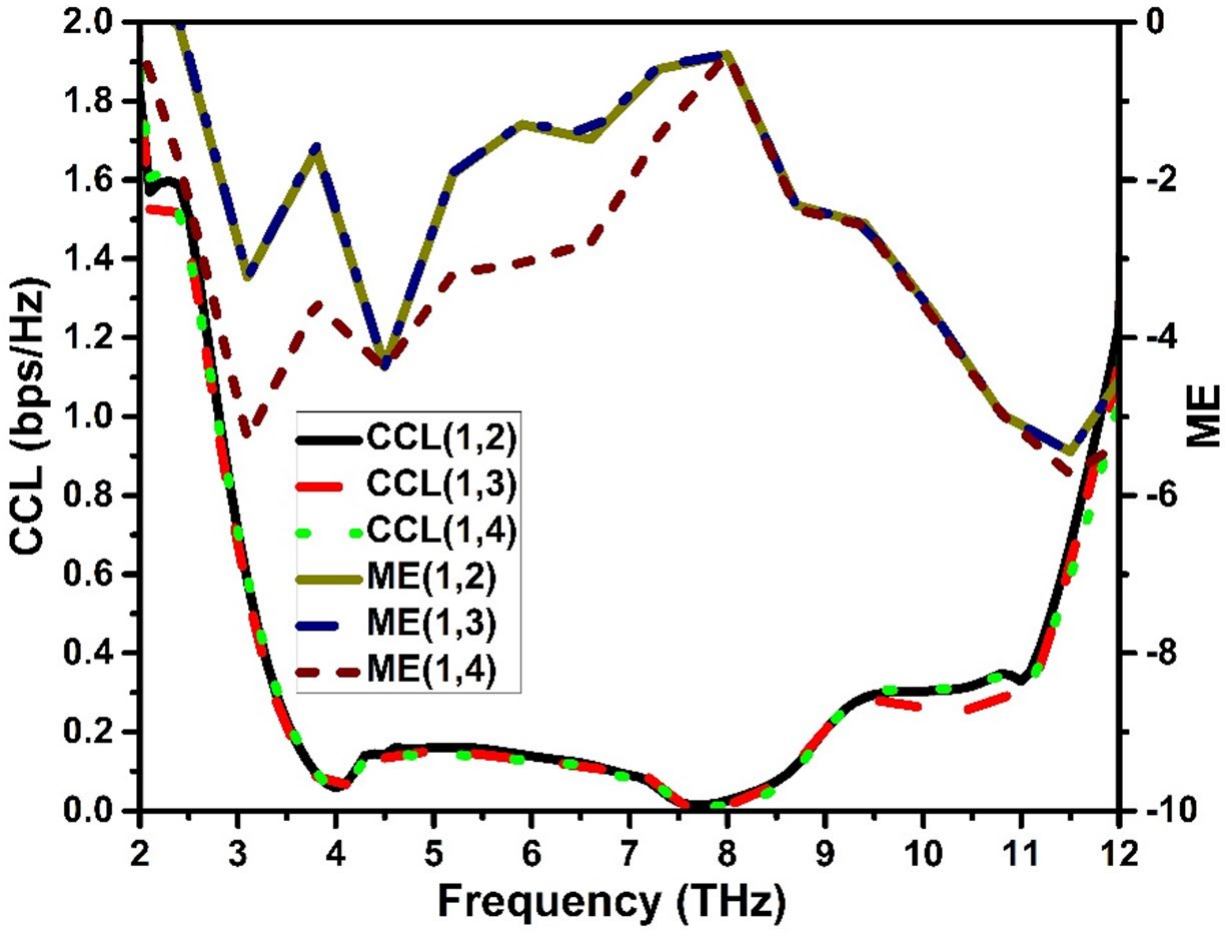
Diversity features: CCL and ME.

The maximum data rate that may be firmly delivered via a MIMO channel is referred to as its capacity. The number of antennas at both ends, the signal-to-noise ratio (SNR), and channel conditions all have an impact on capacity and loss is referred to be channel capacity loss (CCL). The CCL of a MIMO antenna system is a measurement of the highest data rate that can be successfully sent across the communication channel. Antenna correlation measures how closely the signals received by various antennas are connected. High correlation between antennas can lead to a drop in the diversity gain and, subsequently, a loss in channel capacity. The CCL can be computed using Eq. (5). When measuring a MIMO antenna system’s performance in a spatial multiplexing mode, multiplexing efficiency (ME) takes into account correlation and efficiency amongst MIMO radiating elements and is computed using Eq. (6). The CCL and ME of the suggested antenna are displayed in Fig. 12.

5 $$\:CCL=\:-{log}_{2}\mathrm{det}{\left(\alpha\:\right)}^{R}\:\:\:\:\:\:\:$$

where $\:{\alpha\:}^{R}$ is the receiving antenna correlation matrix. For four-element MIMO system, $\:{\alpha\:}^{R}\:$expressed as:

$$\:{\alpha\:}^{R}=\left|\begin{array}{cccc}{\rho\:}_{11}&\:{\rho\:}_{12}&\:{\rho\:}_{13}&\:{\rho\:}_{14}\\\:{\rho\:}_{21}&\:{\rho\:}_{22}&\:{\rho\:}_{23}&\:{\rho\:}_{24}\\\:{\rho\:}_{31}&\:{\rho\:}_{32}&\:{\rho\:}_{33}&\:{\rho\:}_{34}\\\:{\rho\:}_{41}&\:{\rho\:}_{42}&\:{\rho\:}_{43}&\:{\rho\:}_{44}\end{array}\right|$$

$$\:{\rho\:}_{ii}=1-\left|\sum\:_{n=1}^{N}{S}_{in}^{\mathrm{*}}\:{S}_{ni}\right|for\:i,j=\mathrm{1,2},\mathrm{3,4},\dots\:$$

$$\:{\rho\:}_{ij}=1-\left|\sum\:_{n=1}^{N}{S}_{in}^{\mathrm{*}}\:{S}_{nj}\right|for\:i,j=\mathrm{1,2},\mathrm{3,4},\dots\:$$

$$\:ME=\:\sqrt{{\eta\:}_{1}{\eta\:}_{2}(1-{\rho\:}_{ij})}\:\:\:\:\:\:\:\:\:\:\:\:\:\:\:\:\:\:\:\:\:\:\:\:\:\:\:\:\:\:\:\:\:\:\:\:\:\:\:\:\:\:\:\:\:\:\:\:\:\:\:\:\:\:\:\:\:\:\:\:\:\:\:\:\left(6\right)\:\:\:\:$$

where $\:{\eta\:}_{1}{\eta\:}_{2}$ are radiation efficiencies of 1st and 2nd elements.

### Comparative analysis

A numerical comparison of the frequency range, size, bandwidth, and diversity properties of representative THz MIMO antenna designs from the literature with the proposed design is shown in Table 1. The proposed antenna demonstrates consistent electromagnetic and diversity metrics within the investigated THz band under full-wave simulation. It is noted that Ref^12^. operates over a broader fractional bandwidth and lower normalized electrical size (0.36λ × 0.36λ), whereas the present work focuses on CMA-based isolation enhancement and modal behavior within the 3.3–11.1 THz band. This comparison reflects different optimization objectives in THz MIMO antenna design rather than absolute dimensional superiority.

**Table 1:** The performance comparison of the proposed antenna with previously reported THz MIMO antennas.

Ref	Technique	Antenna dimensions (µm^3^)	Antenna dimensions (λ3)	Number of ports	Impedance bandwidth (THz)	Fractional bandwidth	Isolation (dB)
^2^	Orthogonal and FSS	647.5 × 100 × 1	4.97 × 0.77 × 0.008	4	2.3–2.7, 5.5–8.9	16, 47.2	30
^12^	DGS	45 × 45 × 2	0.36 × 0.36 × 0.016	4	2.38–11.8	132	18
^16^	DGS	400 × 150 × 80	0.047 × 0.018 × 0.009	4	0.035–0.2	140	20
^19^	Orthogonal	1325 × 1325 × 2	1.13 × 1.13 × 0.002	4	0.257–0.370	36	15
^22^	DGS	600 × 800 × 10	20 × 26.7 × 0.33	4	10–13, 13.7–18.9, 19.7–20	26, 32, 1.5	15
^24^	DGS	700 × 700 × 55	0.46 × 0.46 × 0.036	4	0.198–0.324	48	12
^25^	reduced ground plane	60 × 60 × 10	1.9 × 1.9 × 0.32	2	9.5–24	86.6	20
Proposed	Hybrid decoupling	46 × 46 × 2	0.51 × 0.51 × 0.022	4	3.3–11.1	108	22

Ref	ECC	DG	MEG	TARC	CCL	ME
^2^	0.001, 0.00001	10	-3	-	0.1	-
^12^	0.18	> 9.4	<-3	<-5	0.3	<-1
^16^	0.025	9.88	-	<-25	0.027	-
^19^	0.01	9.96		<-10	0.35	
^22^	0.35	9.7	-	<-5	0.8	-
^24^	0.05	10	-3	<-5	-	-
^25^	0.01	10	-3	<-10	0.5	-
Proposed	< 0.1	> 9.5	≤-6	<-10	0.2	< 0

*- not available, λ- computed at the lowest frequency.

## Conclusion

A circular patch incorporating a parasitic element with a lowered ground plane is investigated for operation in the wideband THz frequency range. The four-port THz antenna covers the 3.3–11.1 THz frequency range. A decoupling structure comprising NL and DGP reduces mutual coupling between antenna components. High isolation contributes to reduced mutual coupling and stable multi-port operation within the investigated band. By creating its equivalent circuit using lumped parts, the impedance bandwidth of the proposed antenna is verified. The presented antenna exhibits isolation above 22 dB and diversity metrics within commonly accepted MIMO limits under full-wave simulation. The operating band of 3.3–11.1 THz corresponds to wavelengths of approximately 90–27 μm, requiring micrometer-scale fabrication tolerances. Therefore, the current research is confined to the electromagnetic simulation and modal analysis. Future work is on experimental validation and fabrication-based verification. The proposed study provides a CMA-based methodological approach for isolation enhancement in THz MIMO antenna systems.

## Data Availability

The datasets used and/or analyzed during the current study available from the corresponding author on reasonable request.

## References

[1] Sarkar, P., Saha, A., Banerjee, A. & Chakraborty, V. Design and analysis of MIMO antenna array for TeraHertz communication. *J. Opt.*10.1007/s12596-024-01739-8 (2024).

[2] Sharma, M. K., Sharma, A. & Kumari, R. A CPW THz-MIMO antenna with reduced mutual coupling using Frequency Selective Surface for future wireless applications. *Opt. Mater.* **148**, 114929. 10.1016/j.optmat.2024.114929 (2024).

[3] Amraoui, Y., Halkhams, I., El Alami, R., Jamil, M. O. & Qjidaa, H. High isolation MIMO antenna array for multiband terahertz applications. *Results Eng.* **23**, 102842. 10.1016/j.rineng.2024.102842 (2024).

[4] Pant, R. & Malviya, L. THz antennas design, developments, challenges, and applications: A review. *Int. J. Commun Syst*. 10.1002/dac.5474 (2023).

[5] Al-Bawri, A. A. M. S. S., Abdulkawi, W. M., Aljaloud, K. & Islam, M. T. High gain metamaterial-based 3D cross-shaped THz 16-port massive MIMO antenna array for future wireless network. *Opt. Quant. Electron.*10.1007/s11082-023-05584-0 (2023).

[6] Kiani, N., Hamedani, F. T. & Rezaei, P. Graphene-Based Quad-Port MIMO Reconfigurable Antennas for THz Applications. *Silicon* **16** (9), 3641–3655. 10.1007/s12633-024-02939-4 (2024).

[7] Tale, S. et al. Meta Surface-Based Multiband MIMO Antenna for UAV Communications at mm-Wave and Sub-THz Bands, Drones, **8 **(8), 403–403, 10.3390/drones8080403 (2024).

[8] Saxena, G. Csrr and Ebg Loaded Wideband Thz Dielectric Resonator Mimo Antenna for Nano Communication and Bio-Sensing Applications, 10.2139/ssrn.4729472 (2024).

[9] Han, C., Chen, Y., Yan, L., Chen, Z. & Dai, L. Cross Far- and Near-Field Wireless Communications in Terahertz Ultra-Large Antenna Array Systems. *IEEE Wirel. Commun.*10.1109/mwc.003.2300004 (2024).

[10] Bodet, D. M. & Jornet, J. M. Directional Antennas for Sub-THz and THz MIMO Systems: Bridging the Gap Between Theory and Implementation. *IEEE Open. J. Commun. Soc.* **4**, 2261–2273. 10.1109/ojcoms.2023.3318017 (2023).

[11] Abdulkarem, H. M. et al. Design and analysis of split ring resonator engraved metamaterial broadband and high gain patch antenna for THz applications. *Optik* **287**, 171054–171054. 10.1016/j.ijleo.2023.171054 (2023).

[12] Mohanty, A. & Sahu, S. A Micro 4-port THz MIMO antenna for nano communication networks, Photonics and Nanostructures - Fundamentals and Applications, **53**, 101092, 10.1016/j.photonics.2022.101092 (2023).

[13] Kumar, P. et al. Design and analysis of wideband four-port MIMO antenna with DGS as decoupling structure for THz applications. *Results Opt.* **13**, 100573–100573. 10.1016/j.rio.2023.100573 (2023).

[14] Maurya, N. K., Kumari, S., Pareek, P. & Singh, L. Graphene-based frequency agile isolation enhancement mechanism for MIMO antenna in terahertz regime. *Nano Commun. Netw.* **35**, 100436–100436. 10.1016/j.nancom.2023.100436 (2023).

[15] Ammar Armghan, K. et al. Design and Development of Ultrabroadband, High-Gain, and High-Isolation THz MIMO Antenna with a Complementary Split-Ring Resonator Metamaterial. *Micromachines* **14** (7), 1328–1328. 10.3390/mi14071328 (2023). 37512639 10.3390/mi14071328 PMC10386145

[16] Sharma, M. K. & Sharma, A. Compact size easily extendable self isolated multi-port multi-band antenna for future 5G high band and sub-THz band applications. *Opt. Quant. Electron.*10.1007/s11082-022-04313-3 (2022).

[17] None Nishtha, R. S., Yaduvanshi & Varshney, G. Isolation control for implementing the single dielectric resonator based tunable THz MIMO antenna and filter. *Opt. Quant. Electron.*10.1007/s11082-023-04623-0 (2023).

[18] Singh, R. & Varshney, G. Isolation enhancement technique in a dual-band THz MIMO antenna with single radiator. *Opt. Quant. Electron.*10.1007/s11082-023-04811-y (2023).

[19] Pandey, G. K., Thipparaju, R. R. & Mondal, S. Graphene based quad port terahertz MIMO antenna for wireless indoor communications. *Opt. Quant. Electron.*10.1007/s11082-023-05050-x (2023).

[20] Raghunath, J. et al. A Quad-Port Nature-Inspired Lotus-Shaped Wideband Terahertz Antenna for Wireless Applications. *J. Sens. Actuator Networks*. **12** (5), 69–69. 10.3390/jsan12050069 (2023).

[21] Alqahtani, A. S. A triple band high gain THz antenna for satellite, space and terrestrial applications. *Opt. Quant. Electron.*10.1007/s11082-023-05257-y (2023).

[22] Althuwayb, A. A. et al. Broadband, high gain 2 × 2 spiral shaped resonator based and graphene assisted terahertz MIMO antenna for biomedical and WBAN communication. *Wireless Netw.* **30** (1), 495–515. 10.1007/s11276-023-03494-3 (2023).

[23] Babu, K. V. et al. Performance Analysis of a Photonic Crystals Embedded Wideband (1.41–3.0 THz) Fractal MIMO Antenna Over SiO2 Substrate for Terahertz Band Applications. *Silicon* **15** (18), 7823–7836. 10.1007/s12633-023-02622-0 (2023).

[24] Singh, A. K., Mahto, S. K., Kumar, P. & Sinha, R. Analysis of path loss and channel capacity in quad element MIMO antenna for terahertz communication systems. *Int. J. Circuit Theory Appl.*10.1002/cta.3473 (2022).

[25] Patri Upender & Kumar, A. Implementing reconfigurable circularly polarized two port MIMO DRA for THz applications. *Opt. Quant. Electron.*10.1007/s11082-023-05188-8 (2023).

